# DR5 suppression induces sphingosine-1-phosphate-dependent TRAF2 polyubiquitination, leading to activation of JNK/AP-1 and promotion of cancer cell invasion

**DOI:** 10.1186/s12964-017-0174-1

**Published:** 2017-05-08

**Authors:** You-Take Oh, Ping Yue, Shi-Yong Sun

**Affiliations:** Department of Hematology and Medical Oncology, Emory University School of Medicine and Winship Cancer Institute, 1365-C Clifton Road, Clinical Building C3088, Atlanta, GA 30322 USA

**Keywords:** Death receptor 5, Invasion, S1P, Caspase-8, TRAF2, Polyubiquitination

## Abstract

**Background:**

Death receptor (DR5), a well-characterized death domain-containing cell surface pro-apoptotic protein, has been suggested to suppress cancer cell invasion and metastasis. However, the underlying mechanisms have not been fully elucidated. Our recent work demonstrates that DR5 suppression promotes cancer cell invasion and metastasis through caspase-8/TRAF2-mediated activation of ERK and JNK signaling and MMP1 elevation. The current study aimed at addressing the mechanism through which TRAF2 is activated in a caspase-8 dependent manner.

**Results:**

DR5 knockdown increased TRAF2 polyubiquitination, a critical event for TRAF2-mediated JNK/AP-1 activation. Suppression of sphingosine-1-phosphate (S1P) generation or depletion of casapse-8 inhibited not only enhancement of cell invasion, but also elevation and polyubiquitination of TRAF2, activation of JNK/AP-1 activation and increased expression of MMP1 induced by DR5 knockdown.

**Conclusions:**

Both S1P and caspase-8 are critical for TRAF2 stabilization, polyubiquitination, subsequent activation of JNK/AP1 signaling and MMP1 expression and final promotion of cell invasion.

## Background

Death receptor (DR5; also called as TRAIL-R2 or Killer/DR5) is a death domain-containing transmembrane cell surface protein. DR5 is well known to mediate apoptosis upon ligation with its ligand or induction of its clustering or aggregation (e.g., with an agonistic antibody or overexpression). This process involves activated DR5 interaction with the adaptor protein, Fas-associated death domain (FADD), which further recruits and activates caspase-8 [1, 2].

Despite its well characterized apoptotic function, the precise physiological or pathological role of DR5 in the regulation of human cancer development remains unclear [3, 4]. Mice deficient in mouse TRAIL death receptor (mDR; the sole mouse ortholog of human DR4 and DR5) show increased susceptibility to tumorigenesis, such as Myc-driven lymphoma and diethylnitrosamine-induced hepatocarcinogenesis [5]. Moreover, mDR deficiency in mice enhances lymph node metastasis of skin carcinoma [6] and metastasis of lymphoma cells to the liver and lung during c-myc-driven lymphomagenesis [5], suggesting that mDR may be critical for the negative regulation of tumor metastasis. Studies with human cancer samples have shown a reduced DR5 expression in metastatic lesions of melanoma [7] and in primary head and neck tumors with metastasis and their matching lymph node metastasis [8]. Furthermore, inactivating mutations primarily in the death domain of DR5 were detected in 20% of tissues from breast cancer patients with lymph node metastasis, but were not found in tissues from breast cancer patients without metastasis [9]. The DR5 agonistic antibody lexatumumab robustly suppresses lymph node or lung metastasis in an orthotopic model of triple-negative breast cancer [10]. Our recent study with various human cancer cells clearly shows that genetic knockdown or knockout of DR5 significantly increased cancer cell invasion and metastasis in vivo [11]. These findings support the notion that DR5 may be associated with suppression of cancer metastasis.

However, opposing findings have also been reported. One study suggested that oncogenic K-Ras and its effector, Raf1, can convert death receptors (e.g., Fas and DR5) into invasion-inducing receptors by suppressing the ROCK/LIM kinase pathway, and this is essential for K-Ras/Raf1-driven metastasis formation [12]. Another recent publication reports that mDR and human DR5 promote K-Ras-driven cancer progression, invasion and metastasis since deficiency of mDR suppressed tumor growth and metastasis in K-Ras-driven carcinogenesis [13]; however, the results of this group are contradictory to their previous findings using a H-Ras-driven skin carcinogenesis model [6]. Hence, the role of DR5 in the regulation of cancer growth and metastasis remains unclear and needs further investigation.

Our recently study has suggested that, under conditions of DR5 suppression, available FADD and caspase-8 may recruit and stabilize tumor necrosis factor receptor-associated factor 2 (TRAF2), resulting in the activation of ERK and JNK signaling and subsequent AP-1-dependent expression and activation of MMPs (e.g., MMP1) and final promotion of invasion and metastasis of cancer cells [11]. However, the association between caspase-8 and TRAF2 activation is undefined and hence was the focus of the current study.

## Methods

### Reagents

SK1-II was purchased from Echelon Bioscience, Inc (Salt Lake City, UT). The human monoclonal DR5 agonistic antibody, AMG655 (Conatumumab), was supplied by Amgen (Thousand Oaks, CA). Antibodies against K63 and K48 were purchased from Cell Signaling Technology (Danvers, MA). Other antibodies and reagents were the same as described previously [11].

### Cell lines and cell culture

A549, 801C and HEK293T cells were described previously [11]. These cell lines were cultured in RPMI 1640 medium containing 5% fetal bovine serum at 37 °C in a humidified atmosphere of 5% CO_2_ and 95% air.

### Western blot analysis

Whole-cell protein lysates were prepared and analyzed by Western blotting as described previously [14]. Protein levels were quantified with NIH Image J software based on band density and were normalized to an internal loading control protein.

### Expression constructs and transfection

HA-Ubiquitin-wild-type (WT), -K63 and -K48 expression constructs and his-Ubiquitin expression plasmid were purchased from Addgene (Cambridge, MA). WT and mutant (C360A) Caspase-8 expression constructs [15] were provided by Dr. K. Vuori (Burnham Institute for Medical Research, La Jolla, CA). Flag-TRAF2 expression construct [16] was provided by Dr. H. Habelhah (University of Iowa, Iowa City, IA). Generally, cells were transfected with the given plasmids using Lipofectamine™ 2000 (Invitrogen) as instructed by the manufacturer’s protocol.

### Gene silencing using small interfering siRNA (siRNA) or short hairpin RNA (shRNA)

Gene silencing was achieved by either transfecting siRNA using HiPerFect transfection reagent (Qiagen, Valencia, CA) following the manufacturer’s instructions or infecting cells with lentiviruses harboring a given shRNA. Control (i.e., non-silencing) and DR5-specific siRNAs were described previously [14]. DR5 shRNA in pLKO.1 (TRCN0000005929) was purchased from Open Biosystems (Huntsville, AL). Caspase-8 (sc-29930) and sphingosine kinase 1 (SphK1; sc-44114) siRNAs were purchased from Santa Cruz Biotechnology, Inc. Gene silencing effects were evaluated by Western blot analysis as described above.

### Reporter plasmids, transient transfection, and luciferase activity assay

AP-1 (pAP1-luc) and MMP1 promoter luciferase reporter constructs were described previously [11]. Transient co-transfection of DR5 siRNA, Flag-TRAF2 and pCH110, a plasmid expressing β-galactosidase (β-gal) with lipefectamine was conducted in 24-well plates followed by luciferase assays as described previously [17]. Luciferase activity was normalized to β-gal activity, which was measured as described previously [18].

### Immunoprecipitation (IP)

The cells were lysed in RIPA buffer with protease and phosphatase inhibitors. The cell lysates were then incubated with anti-Flag M2, anti-HA agarose or anti-TRAF2 (sc-7187; Santa Cruz Biotechnology, Inc) at 4 °C overnight according to the manufacturer’s instruction (for tagged proteins). The beads were then washed four times (5 min each) with the same buffer used for cell lysis and boiled in 2 × SDS sample buffer for 5 min. Samples were then analyzed by SDS-PAGE followed by Western blotting.

### Cell invasion and growth assays

Measurements for cell invasion and cell numbers were the same as described previously [11].

### Statistical analyses

The statistical significance of differences between two groups was analyzed with two-sided unpaired Student's *t* tests when the variances were equal or with Welch’s corrected *t* test when the variances were not equal by use of Graphpad InStat 3 software (GraphPad Software, San Diego, CA).

## Results

### DR5 knockdown increases TRAF2 polyubiquitination that is important for activation of JNK/AP-1 signaling

**Fig. 1:**
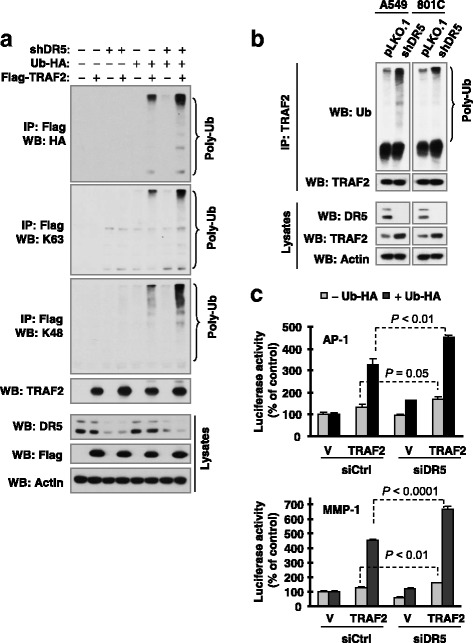
DR5 knockdown increases TRAF2 polyubiquitination (**a** and **b**), which enhances AP-1 transactivation (**c**), **a**, HEK293T cells were co-transfected with the indicated plasmids carrying the indicated genes. After 42 h, the cells were lysed for IP with anti-Flag antibody and subsequent Western blotting (WB) for the indicated proteins. The experiments were done twice with identical results. **b**, Whole-cell protein lysates were prepared from the indicated different cell lines and then subjected to IP with TRAF2 antibody and subsequent Western blotting (WB) for different proteins as indicated. **c**, HEK293T cells were co-transfected with vector (V) or Flag-TRAF2 plasmid and an control (siCtrl) or DR5 siRNA (siDR5) together with an MMP1 or AP-1 luciferase reporter construct and pCH110 plasmid. After 36 h, the cells were lysed to assay luciferase activity, which was normalized to β-gal activity. Each column represents a mean ± SD of triplicate determinations from a representative experiment. The experiments were done twice with similar results

In our previous report, we have shown that DR5 knockdown elevated TRAF2 levels and increased AP-1, but not NF-κB, activity [11]. It has been suggested that TRAF2 polyubiquitination, including K63 and K48 polyubiquitination, is required for TRAF2 to activate JNK, but not NF-κB [19]. Therefore, we first determined whether DR5 regulates TRAF2 polyubiquitination. As presented in Fig. 1a, co-transfection of Flag-TRAF2 and Ub-HA led to increased levels of polyubiquitinated TRAF2 including K63- and K48-specific ubiquitination, which were further increased by DR5 knockdown. In DR5-knocked down A549 and 801C cell lines, increased endogenous TRAF2 polyubiquitination was also detected in comparison with pLKO.1 controls cells (Fig. 1b). These data together suggest that DR5 knockdown increases TRAF2 polyubiquitination. Moreover, we tested whether TRAF2 polyubiquitination affects AP-1 activity. Co-transfection of Flag-TRAF2 and Ub-HA was much more effective than TRAF2 alone in increasing MMP1 (carrying AP-1 binding site) and AP-1 promoter activity. When DR5 was knocked down, these effects were further significantly enhanced (Fig. 1c). These results suggest that TRAF2 polyubiquitination indeed enhances AP-1 transactivation.

### DR5 activation by an agonistic antibody promotes TRAF2 degradation, decreases TRAF2 polyubiquitination, suppresses JNK signaling and inhibits invasion

**Fig. 2:**
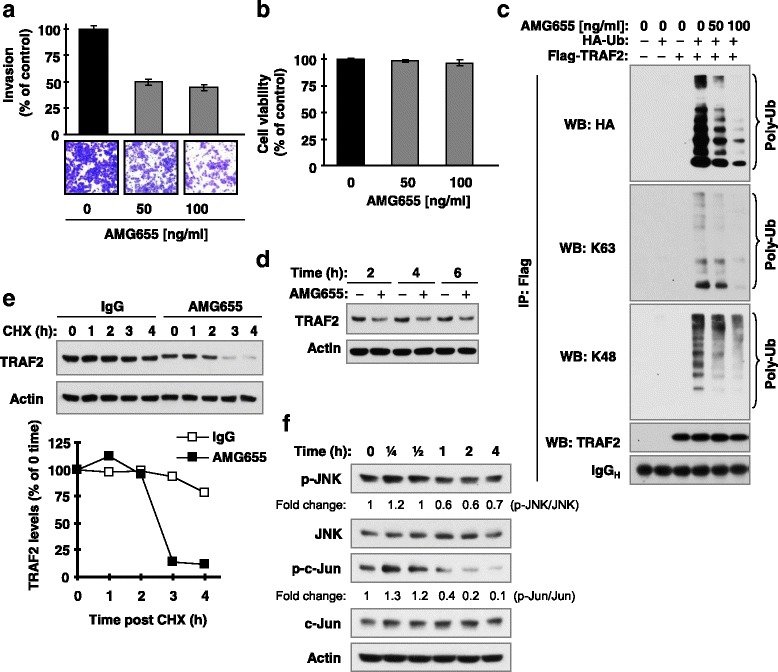
The DR5 agonistic antibody, AMG655, at concentration ranges that suppress invasion (**a** and **b**), suppresses TRAF2 polyubiquitination (**c**), enhances TRAF2 degradation (**d** and **e**) and causes delayed inhibiton of JNK signaling (**f**). **a** and **b**, A549 cells were allowed to invade through transwells coated with Matrigel for 48 h in the bottom well containing the indicated concentration of AMG655. Invaded cells at the lower surface were then stained and quantified (**a**). Under the tested condition, AMG655 minimally affected cell survival (**b**). **c**, HEK293T cells were co-transfected with Flag-TRAF2 and HA-Ub for 30 h and then stimulated with different doses of AMG655 as indicated for additional 90 min. The cells were then harvested for preparation of whole-cell protein lysates, IP and subsequent Western blotting (WB) for the indicated proteins. **d**-**f**, A549 cells were exposed to 100 ng/ml AMG655 for the given times (**d** and **f**). Moreover, A549 cells were pre-exposed to 100 ng/ml AMG655 for 2 h followed by treatment with 10 μg/ml CHX for additional times as indicated (**e**). After these treatments, the cells were harvested for preparation of whole-cell protein lysates and subsequent Western blot analysis. Protein levels were quantified with NIH Image J Software and were normalized to actin. The results were plotted as relative TRAF2 levels compared to those at time 0 of CHX treatment ((**e**); *bottom panel*)

Next we checked the impact of DR5 activation with an agonistic antibody on TRAF2 polyubiquitinaiton. With the concentration ranges that minimally affected cell viability (Fig. 2b), the DR5 agonistic antibody AMG655 significantly reduce the invasion of cancer cells (Fig. 2a). In contrast to DR5 knockdown, AMG655 substantially suppressed TRAF2 polyubiquitination including K63- and K48-specific ubiquitinations, in a concentration-dependent manner (Fig. 2c). Moreover we found that AMG655 decreased TRAF2 levels and enhanced TRAF2 degradation rate (Figs. 2d and e), suggesting that AMG655 destabilizes TRAF2 protein. We also observed that AMG655 caused a delayed reduction of p-JNK and p-c-Jun levels after a transient elevation (Fig. 2f). Collectively, we suggest that AMG655-induced DR5 activation promotes TRAF2 degradation accompanied with a suppression of polyubiquitinaiton and JNK signaling, resulting in eventual suppression of cancer cell invasion.

### Sphingosine-1-phosphate (S1P) participates in DR5 knockdown-induced promotion of cell invasion

**Fig. 3:**
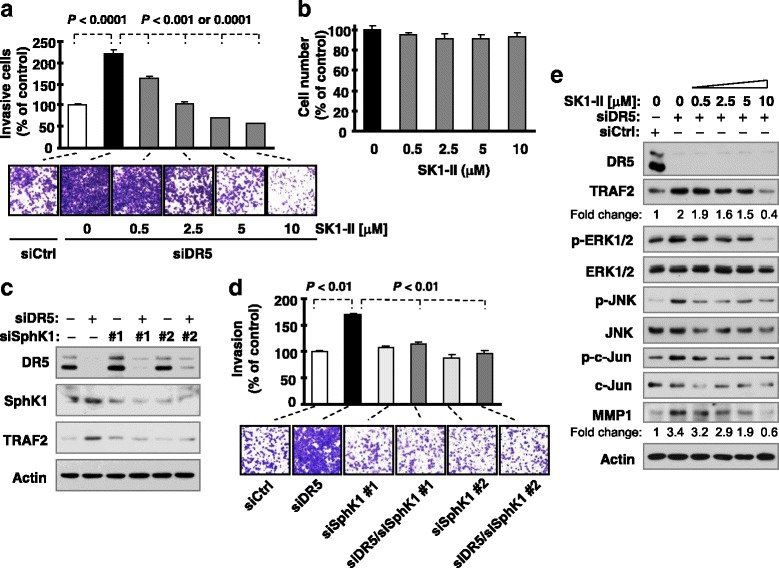
Inhibition of S1P generation or function with SK1-II (**a** and **b**) and SphK1 knockdown (**c** and **d**) attenuates DR5 silencing-induced cell invasion accompanied with blockage of DR5 knockdown-induced activation of JNK and ERK signaling and elevation of TRAF2 and MMP1 (**e**). **a** and **b**, A549 cells transfected with control (Ctrl) or DR5 siRNA were plated after 12 h in Matrigel invasion chambers for cell invasion assays and then exposed to the given concentrations of SK1-II in the bottom wells for an additional 36 h. The invading cells were stained, photographed and measured (**a**). Moreover A549 cells were seeded in 96-well plates and exposed to different concentrations of SK1-II for about 48 h, and cell numbers were measured with the MTS assay (**b**). The data are means ± SDs of triplicate determinations. **c** and **d**, A549 cells transfected with the indicated siRNAs alone or in combinations were seeded in 12-well plates for Western blotting to detect the given proteins (**c**) and in Matrigel invasion chambers for cell invasion assays (**d**) after approximately 48 h incubation. The data are means ± SDs of duplicate determinations from a representative experiment. The experiments were conducted 2-3 times with similar results. **e**, A549 cells were transfected with control (Ctrl) and DR5 siRNA and after 24 h were exposed to different doses of SK1-II as indicated for an additional 15 h. The cells were then subjected to preparation of whole-cell protein lysates and subsequent Western blot analysis for the indicated proteins

It has been suggested that S1P specifically binds to TRAF2 and regulates its biological functions (e.g., E3 ligase activity) [20, 21]. Thus, we determined whether S1P contributes to TRAF2-mediated promotion of cell invasion induced by DR5 knockdown. SphK1 is one of the enzymes responsible for the phosphorylation of sphingosine to generate S1P inside cells [22]. Accordingly, SphK1 inhibitors such as SK1-II will decrease intracellular S1P levels. The presence of SK1-II, at concentration ranges that minimally affected cell growth (0.5-10 μM), dose-dependently suppressed cell invasion induced by DR5 knockdown (Figs. 3a and b). Similar results were also generated with direct silencing of SphK1 (Figs. 3c and d).

### S1P contributes to DR5 suppression-induced elevation of TRAF2 and MMP1 and activation of ERK and JNK signaling

We then determined the involvement of S1P in mediating DR5 knockdown-induced activation of ERK and JNK signaling and activation of TRAF2 and MMP1, required events for DR5 suppression-induced enhancement of invasion [11]. We found that inhibition of S1P with SK1-II blocked the increase in the levels of TRAF2, p-ERK1/2, p-JNK, p-c-Jun and MMP1 induced by DR5 knockdown (Fig. 3e). We also noted that silencing of SphK1 prevented TRAF2 elevation induced by DR5 knockdown (Fig. 3c). These results indicate that inhibition of S1P blocks elevation of TRAF2 and MMP1 and activation of JNK and ERK1/2 signaling. These data again support the involvement of S1P in the promotion of cell invasion induced by DR5 knockdown.

### SphK1/S1P signaling is involved in the regulation of TRAF2 polyubiquitination induced by DR5 knockdown

**Fig. 4:**
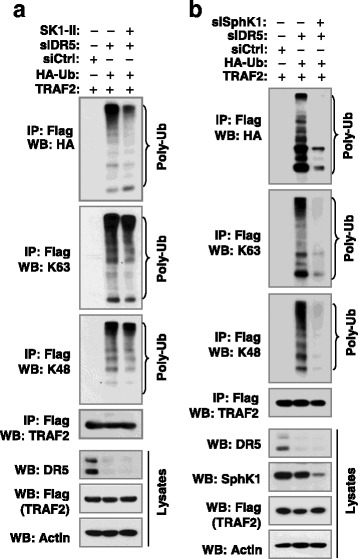
S1P signaling impacts DR5 knockdown-induced TRAF2 polyubiquitination. ***a*** HEK293T cells were co-transfected with the indicated genes or siRNA and after 24 h, were treated with 5 μM SK1-II for an additional 10 h. **b** HEK293T cells were co-transfected with the indicated genes or siRNA and then incubated for 48 h. After the above treatments, whole-cell protein lysates were then prepared from these cells and subjected to IP and subsequent Western blotting (WB) for the indicated proteins

Given that S1P binds to TRAF2 and regulates E3 ubiquitin ligase activity, particularly for K63 polyubiquitination [20], we further determined the role of SphK1/S1P signaling in the regulation of TRAF2 polyubiquitination induced by DR5 knockdown. The presence of SKI-II reduced the levels of polyubiquitinated TRAF2 induced by DR5 knockdown (Fig. 4a). Consistently, we detected large amounts of polyubiquitinated TRAF2 in cells transfected with DR5 siRNA, but minimal levels in cells co-transfected with DR5 and SphK1 siRNAs (Fig. 4b). These results clearly show that SphK1/S1P signaling is required for DR5 knockdown-induced enhancement of TRAF2 polyubiquitination.

### Caspase-8 regulates TRAF2 polyubiquitination

**Fig. 5:**
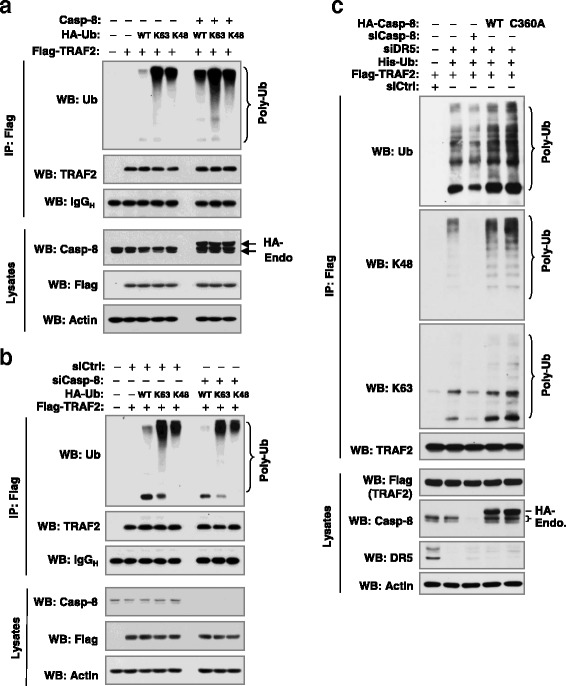
Caspase-8 modulates TRAF2 polyubiquitination (**a** and **b**) independent of its caspase activity (**c**). **a**, HEK293T cells were co-transfected with TRAF2, caspase-8 and WT or mutant Ub plasmid as indicated. **b**, HEK293T cells were co-transfected with caspase-8 siRNA and plasmids carrying the indicated genes. **c**, HEK293T cells were co-transfected with Flag-TRAF2 and other indicated genes or siRNAs. After 34 h of the above transfections, whole-cell protein lysates were prepared from these cells and subjected to IP and subsequent Western blotting (WB) for the indicated proteins

In our previous study, we showed that caspase-8 is important for TRAF2 accumulation, activation of ERK1/2 and JNK/AP-1 signaling and promotion of invasion induced by DR5 knockdown [11]. Here, we asked whether caspase-8 is also involved in the regulation of TRAF2 polyubiquitination. Hence, we compared TRAF2 polyubiquitination in the absence and presence of caspase-8. We detected ubiquitinated TRAF2 in cells co-transfected with TRAF2 and WT, particularly K63 or K48 ubiquitin expression plasmids. The levels of these ubiquitinated proteins were substantially enhanced when caspase-8 was co-expressed (Fig. 5a). In contrast, when endogenous caspase-8 was depleted by transfection with caspase-8 siRNA, the levels of these ubiquitinated proteins were diminished (Fig. 5b). These results indicate that caspase-8 regulates TRAF2 polyubiquitination.

We also determined whether caspase-8 activity is required for regulation of DR5 knockdown-induced TRAF2 polyubiquitination. While knockdown of caspase-8 reduced TRAF2 polyubiquitination induced by DR5 knockdown, enforced expression of both WT and mutant (C360A) caspase-8 elevated TRAF polyubiquitination induced by DR5 siRNA with comparable potencies (Fig. 5c). Since C360A mutation in the caspase domain of caspase-8 abolishes caspase activity [15], we suggest that caspase activity is not required for caspase-8 to modulate DR5 knockdown-induced TRAF2 polyubiquitination.

## Discussion

Involvement of TRAF2 in the positive regulation of cancer cell invasion has been suggested in some previous studies [11, 23, 24]. TRAF2 overexpression has been documented in tumor samples from certain types of cancer such as human pancreatic cancer, breast cancer and gastric cancer, and is associated with cancer progression, metastasis, and shorter patient survival [23, 25, 26]. The current study continues our previous exploration of the critical role of TRAF2-dependent enhancement of cancer cell invasion and metastasis induced by DR5 suppression [11] to further understand the mechanism by which TRAF2 is activated during DR5 suppression-induced promotion of cancer cell invasion.

TRAF2 is known to mediate the activation of both JNK/AP-1 and NF-κB albeit through distinct mechanisms [27, 28]. However, TRAF2 polyubiquitination, including both K63 and K48 ubiquitination, is required for TRAF2 to activate JNK, but not NF-κB [19]. Knockdown of DR5 primarily activates JNK/AP-1 signaling, but not NF-κB as demonstrated in our previous report [11]. In this study, we clearly showed that DR5 knockdown increases the polyubiquitination of TRAF2 including both K63 and K48 polyubiquitination (Fig. 1a and b). Moreover, ubiquitination of TRAF2 enhanced AP-1 and MMP1 transcriptional activity including DR5 knockdown-induced transactivation of AP-1 and MMP1 (Fig. 1c). Complementarily, the DR5 agonistic antibody, AMG655, at low concentration ranges that minimally affect cell viability, suppressed TRAF2 polyubiquitination accompanied with enhanced TRAF2 protein degradation and delayed suppression of JNK signaling (Fig. 2). Taken together, these data suggest that TRAF2 polyubiquitination plays an important role in mediating DR5-dependent modulation of cancer cell invasion. Moreover, we have shown that caspase-8 positively regulates TRAF2 polyubiquitination, since enforced expression of ectopic caspase-8 enhanced TRAF2 polyubiquitination, whereas knockdown of endogenous caspase-8 expression diminished TRAF2 polyubiquitination (Figs. 5a and b). In agreement with our previous finding that caspase-8 enzymatic activity is not required for mediating DR5 suppression-induced cancer cell invasion [11], the current study further demonstrates that caspase-8 mediates TRAF2 polyubiquitination induced by DR5 suppression independent of its caspase activity (Fig. 5d).

S1P is a pleiotropic lipid mediator that regulates cell growth, cell survival, cell invasion, vascular maturation, and angiogenesis, processes that are important for cancer progression [22]. Although the involvement of S1P in promoting cancer cell invasion and metastasis has been documented in various types of cancers including ovarian, esophageal, prostate, hepatocellular, head and neck, renal, colorectal, breast and pancreatic cancers, glioblastoma and Wilms tumor [29–40], the underlying mechanisms are largely unclear. It is known that S1P exerts most of its biological actions as a specific ligand for a family of five cognate G protein-coupled receptors in addition to its intracellular functions [22]. S1P has also been suggested to specifically bind to TRAF2 and regulates its biological functions (e.g., E3 ligase activity) [20]. Hence S1P is an essential cofactor for TRAF2 biological activity [21]. We found that inhibition of S1P generation or function with SK1-II or SphK1 knockdown suppressed the enhanced invasion of cancer cells induced by DR5 knockdown, blocked DR5 knockdown-induced activation of ERK1/2 and JNK/AP-1 signaling including elevation of TRAF2 and MMP1, and attenuated DR5 knockdown-induced TRAF2 polyubiquitination (Figs. 3 and 4). Therefore, it appears that S1P is involved in TRAF2-dependent activation of ERK1/2 and JNK/AP-1 signaling and promotion of cell invasion induced by DR5 inhibition. We assume that S1P mediates these processes through binding to TRAF2 independent of S1P receptors although this assumption needs further experimental validation. Our current findings in this regard not only support the role of S1P in the positive regulation of cancer cell invasion and metastasis, but also provide insights into the biology accounting for S1P-dependent promotion of cancer cell invasion and metastasis.

**Fig. 6:**
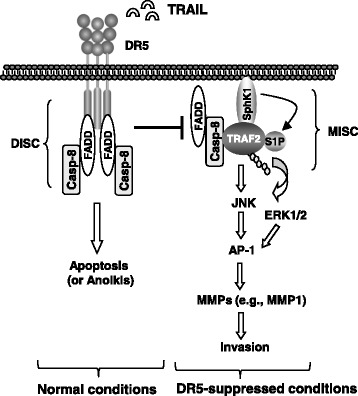
A working model for DR5-mediated suppression of cancer cell invasion. The primary function of DR5 is to mediate apoptosis upon activation through formation of the DISC; this will restrict the formation of another complex, the metastasis and invasion signaling complex (MISC), and subsequently suppress cell invasion. When DR5 is inhibited, available FADD and caspase-8 may recruit and stabilize TRAF2 with the help of S1P, resulting in enhanced TRAF2 polyubiquitination and activation, likely through a self-ubiquitination mechanism. This will further lead to the activation of ERK and JNK signaling and subsequent AP-1-dependent expression and activation of MMPs (e.g., MMP1) and finally, promotion of invasion and metastasis of cancer cells

Considering our previous [11] and current findings, we propose a working model as follows: the activation of DR5 favors formation of the death-inducing signaling complex (DISC), resulting in induction of apoptosis or anoikis as well as other potential biological consequences; this not only leads to the direct killing of detached cancer cells (e.g., via anoikis or TRAIL/DR5-mediated immunosurveillance), but also restricts the formation of the metastasis and invasion signaling complex (MISC), eventually resulting in suppression of cancer cell invasion and metastasis. When DR5 is inhibited, cancer cells will be resistant to anoikis or immunosurveillance. Available FADD and caspase-8 may recruit and stabilize TRAF2; this process will be enhanced by intracellular S1P (e.g., generated by SphK1). Consequently, TRAF2 will be polyubiquitinated and activated, likely through a self-ubiquitination mechanism, resulting in the activation of ERK1/2 and particularly JNK signaling and subsequent AP-1-dependent expression and activation of MMPs (e.g., MMP1) and finally, promotion of invasion and metastasis of cancer cells (Fig. 6).

## Conclusions

The current study has demonstrated that S1P-dependent TRAF2 polyubiquitination, downstream of caspase-8, is important for mediating DR5 suppression-induced promotion of cancer cell invasion. Together with our previous findings [11], we have highlighted a novel mechanism accounting for enhancement of cancer cell invasion and metastasis caused by DR5 suppression.

## Abbreviations

DR5: Death receptor 5
FADD: Fas-associated death domain
IP: Immunoprecipitation
mDR: Murine death receptor
S1P: Sphingosine-1-phosphate
shRNA: Small hairpin RNA
siRNA: Small interfering RNA
SphK2: Sphinogosine kinase 1
TRAF2: Tumor necrosis factor receptor-associated factor 2
WT: Wild-type
